# Acute and delayed sulfur mustard toxicity; novel mechanisms and future studies

**DOI:** 10.2478/v10102-010-0027-x

**Published:** 2010-11

**Authors:** Ahmet Korkmaz, Dun-Xian Tan, Russel J. Reiter

**Affiliations:** 1 Department of Physiology, School of Medicine, Gulhane Military Medical Academy, Ankara, Turkey; 2 Department of Cellular and Structural Biology, The University of Texas Health Science Center at San Antonio, San Antonio, TX, USA

**Keywords:** sulfur mustard, acute toxicity, delayed toxicity, melatonin, epigenetic aberrations

## Abstract

Sulfur mustard (SM), also known as mustard gas, has been the most widely used chemical weapon. The toxicity of SM as an incapacitating agent is of much greater importance than its ability to cause lethality. Acute toxicity of SM is related to reactive oxygen and nitrogen species, DNA damage, poly(ADP-ribose) polymerase activation and energy depletion within the affected cell. Therefore melatonin shows beneficial effects against acute SM toxicity in a variety of manner. It scavenges most of the oxygen- and nitrogen-based reactants, inhibits inducible nitric oxide synthase, repairs DNA damage and restores cellular energy depletion. The delayed toxicity of SM however, currently has no mechanistic explanation. We propose that epigenetic aberrations may be responsible for delayed detrimental effects of mustard poisoning. Epigenetic refers to the study of changes that influence the phenotype without causing alteration of the genotype. It involves changes in the properties of a cell that are inherited but do not involve a change in DNA sequence. It is now known that in addition to genetic mutations, epimutations can also involve in the pathogenesis of a variety of human diseases. Several actions of melatonin are now delineated by epigenetic actions including modulation of histone acetylation and DNA methylation. Future studies are warranted to clarify whether epigenetic mechanisms are involved in pathogenesis of delayed sulfur mustard toxicity and melatonin alleviates delayed toxicity of this warfare agent.

## Introduction

Among the available chemical warfare agents, sulfur mustard (SM), also known as mustard gas, has been a widely used chemical weapon. Because of its devastating toxicity, its use during the World War I earned it the sobriquet “king of the battle gases”. Other compounds such as nitrogen mustard (HN2) were developed during World War II, but found to be unsuitable as a munition. Soon after discovering HN2, it became the first non-hormonal agent used in cancer chemotherapy. A number of HN2 derivatives including cyclophosphamide (CP), ifosfamide, mechlorethamine, melphalan and chlorambucil are valuable cytotoxic and radiomimetic agents for the treatment of cancer (Kehe and Szinicz, 2005).

## Summary of Acute SM Toxicity

Acute toxicity of SM shares almost the same pathophysiologic mechanisms with other toxic agents including CP, paraquat, acetaminophen and doxorubicin. Recent data consistently proves that reactive oxygen species (ROS) (Ozcan *et al*., 2005), nitric oxide (NO·) (Korkmaz *et al*., 2003) produced by inducible nitric oxide synthase (iNOS) (Oter *et al*., 2004), and most importantly peroxynitrite (ONOO^–^) (Korkmaz *et al*., 2005; Yaren *et al*., 2007) are involved in initial detrimental effects of all mustards (Korkmaz *et al*., 2007; Korkmaz *et al*., 2006).

ONOO^–^ is *per se* not a radical but is a powerful nitrosating agent. ONOO^–^ interacts with and covalently modifies all major types of biomolecules including membrane lipids, thiols, proteins and DNA. ONOO^–^ activates matrix metalloproteinases (MMPs) and triggers the expression of selectins and cellular adhesion molecules, via enhancing nuclear factor (NF)-κB and activator protein (AP)-1 activation, thereby promoting pro-inflammatory responses including most importantly tumor necrosis factor (TNF)-α and interleukin (IL)-1β.

ONOO^–^ also induces apoptosis and necrosis in cells depending on the exposure concentration. In case of higher concentration, a DNA repair enzyme poly (ADP ribose) polymerase-1 (PARP-1), mediates ONOO^–^-induced necrosis (Korkmaz *et al*., 2006). PARP-1 detects and signals DNA strand breaks induced by a variety of genotoxic insults. PARP transfers ADP-ribose units from the respiratory coenzyme nicotinamide adenine dinucleotide (NAD^+^) to various nuclear proteins. In case of severe DNA injury, overactivation of PARP-1 depletes the cellular stores of NAD^+^, an essential cofactor in the glycolytic pathway, the tricarboxylic acid cycle, and the mitochondrial electron transport chain. As a result, the loss of NAD^+^ leads to a marked reduction in the cellular pools of ATP, resulting in cellular dysfunction and cell death via the necrotic pathway. Experimental evidence has established that the PARP-1 pathway of cell death plays a pivotal role in tissue injury and organ dysfunction in mustard-induced acute toxicity (Kehe *et al*., 2007; Korkmaz *et al*., 2008).

## Beneficial Effects of Melatonin Against Acute SM Toxicity

There is a large body of evidence that melatonin is major scavenger of both oxygen and nitrogen based radicals including ONOO^–^. Several metabolites of this indolamine also have the capability to detoxify free radicals and their derivatives (Tan *et al*., 2007). Melatonin, possesses genomic actions and regulates the expression of several genes including those for SOD and GSH-Px. Melatonin influences both antioxidant enzyme activity and cellular mRNA levels for these enzymes under both physiological conditions and during elevated oxidative stress (Reyes-Toso *et al*., 2004). These two features in a single molecule are unique for an antioxidant and both actions protect against pathologically-produced free radicals after SM exposure.

In many inflammatory processes, ONOO^–^ rather than oxygen-based radicals is the predominant molecule which decides the fate of cells. Once formed, ONOO^–^ cannot be scavenged by conventional antioxidants. As a multifunctional antioxidant, however, melatonin and its metabolites have unique features over the usual antioxidants including iNOS inhibition and ONOO^–^ scavenging properties against mustard-induced acute toxicity (Sadir *et al*., 2007; Topal *et al*., 2005; Ucar *et al*., 2007; Yildirim *et al*., 2004). Melatonin has been shown to ameliorate inflammation by blocking transcriptional factors and pro-inflammatory cytokines (Mei *et al*., 2002; Sasaki *et al*., 2002; Wang *et al*., 2004) and preserves cellular energy production and ATP level in several pathologic circumstances (Dugo *et al*., 2001; Lopez *et al*., 2006; Tan *et al*., 2005). Thus, melatonin is the only medically suitable versatile antioxidant and anti-inflammatory agent which defeats the cells against all levels of acute mustard toxicity.

## Proposed Mechanism of Delayed SM Toxicity

Unfortunately, it is not clear how mustard gas causes severe multi-organ damage years after even a single exposure (Balali-Mood and Hefazi, 2006). Most metabolites of mustard agents are excreted in the urine within a few weeks after exposure and they do not accumulate within the cells (Somani and Babu, 1989). Cellular acute effects of mustards and several other drugs including acetaminophen and doxorubicin disappear after the exposure ceases. SM is the only warfare agent which has severe delayed effects and causes progressive incapacitation of victims. 34 000 Iranians have been examined 13–20 years after exposure to SM, and it was found that lungs (42.5%), eyes (39%), and skin (24.5%) of victims are affected and these pathologies are almost incurable even with extensive treatments (Khateri *et al*., 2003).

A possible explanation of the delayed mechanism would be epigenetic perturbations caused by SM even after single exposure. The term epigenetic describes the study of inheritable alterations in gene expression that occur in the absence of changes in genome sequence. This is in contrast to genetics, which deals with the transmission of information based on differences in DNA sequence. Therefore, epigenetic gene regulation requires molecular mechanisms that encode information in addition to the DNA base sequence and can be propagated through mitosis and meiosis. Our current understanding of epigenetic regulation of gene expression involves basically two classes of molecular mechanisms: histone modifications and DNA methylation. A variety of enzymes are involved in this process including most importantly histone deacetylases (HDACs), histone acetyl transferases (HATs) and DNA methyltransferases (DNMTs) (Miremadi *et al*., 2007). Alteration of the structure of chromatin is critical to the regulation of gene expression. Chromatin is made up of nucleosomes, which are particles consisting of DNA associated with an octomer of two molecules each of the core histone proteins (H2A, H2B, H4 and H4), around which 146 base pairs of DNA are wound. In resting conditions, DNA is wound tightly around these basic core histones, excluding the binding of the enzyme RNA polymerase II, which activates the formation of messenger RNA. This conformation of the chromatin structure is described as closed, and is associated with the suppression of gene expression.

DNA methylation is another regulation, in which a cytosine base is modified by a DNMT at the C5 position of cytosine, a reaction that is carried out by various members of a single family of enzymes. CpG islands are CG-rich sequences located near coding sequences and serve as promoters for the associated genes and methylation of CpG sites is maintained by DNMTs. DNA methylation is commonly associated with gene silencing and contributes to transcriptional regulation of tissue-specific genes during cellular differentiation. The methylation status of CpG islands within promoter sequences works as an essential regulatory element by modifying the binding affinity of transcription factors to DNA binding sites. Gene transcription only occurs when the chromatin structure is opened up, with unwinding and properly methylated of DNA so that RNA polymerase II and basal transcription complexes can now bind to the naked DNA to initiate transcription.

The epigenotype can be transmitted from a parent cell to a daughter cell maintaining a specific epigenotype within cell lineages. Thus, the phenotype is a result of the genotype, the specific DNA sequence, and the epigenotype. The genotype must exist in a particular chromatin configuration, the epigenotype, which allows a secondary level of fine control over gene expression. The epigenotype shows far greater plasticity than the genotype, and it has been speculated that epigenetic errors could be a major contributor to human diseases (Jiang *et al*., 2004). Epigenotype is generally accepted as being less stable than the genetic system, and more sensitive to chemical toxicants (Bombail *et al*., 2004; McLachlan *et al*., 2001). SM may perturb the epigenetic environment of transcription factors such as NF-κB and AP-1 and/or pro-inflammatory genes such as TNF-α and ILs.

## Lessons-learned from Treatment of Patients with Chronic Obstructive Pulmonary Diseases

One of the major problems in the treatment of chronic obstructive pulmonary diseases (COPD) is glucocorticoid resistance. Although inhaled glucocorticoids are highly effective in asthma, they provide relatively little therapeutic benefit in COPD, despite the fact that active airway and lung inflammation is present. This may reflect that the inflammation in COPD is not suppressed by glucocorticoids, with no reduction in inflammatory cells, cytokines or proteases in induced sputum even with high doses of inhaled and oral glucocorticoids (Loppow *et al*., 2001). Furthermore, histological analysis of peripheral airways of patients with severe COPD shows an intense inflammatory response, despite treatment with high doses of inhaled glucocorticoids (Hogg *et al*., 2004). There is increasing evidence for an active steroid resistance mechanism in COPD, as glucocorticoids fail to inhibit cytokines (e.g., ILs and TNF-α) that they normally suppress.

The predominant effect of glucocorticoids is to switch off multiple inflammatory genes (encoding cytokines, chemokines, adhesion molecules and inflammatory enzymes) that have been activated during the chronic inflammatory process. The increased expression of most of these inflammatory proteins is regulated at the level of gene transcription through the activation of pro-inflammatory transcription factors, such as nuclear NF-κB and AP-1. The molecular pathways involved in regulating inflammatory gene expression are now being delineated and it is now clear that chromatin remodeling and a variety of epigenetic mechanisms play a critical role in the transcriptional control of genes. Stimuli that switch on inflammatory genes do so by changing the chromatin structure of the inflammatory gene, whereas glucocorticoids reverse this process.

Glucocorticoids produce their effect on responsive cells by activating the glucocorticoid receptor (GR) to directly or indirectly regulate the transcription of target genes. Most of the anti-inflammatory actions of glucocorticoids are due to suppression of the actions of AP-1 and NF-κB (Barnes, 2006). The activated GR may directly bind to nuclear receptor co-activators (e.g., p300/CBP) to inhibit their HAT activity, thus preventing the subsequent histone acetylation and chromatin remodeling and leads to inhibition of AP-1 and NF-κB-induced pro-inflammatory gene expression such as TNF-α, IL-1β and adhesion molecules (Adcock *et al*., 2004). Another mechanism is to reverse this process by deacetylating the hyper-acetylated histones through the recruitment of HDAC-2 to the activated co-activator complex (Ito *et al*., 2006). This process results in rewinding and compaction of DNA, exclusion of RNA polymerase, and suppression of inflammatory gene transcription. This mechanism could account for the anti-inflammatory effect of glucocorticoids in inflammatory diseases (Adcock *et al*., 2004).

Patients with COPD has been shown to have a progressive reduction in total HDAC activity that reflects the severity of the disease (Ito *et al*., 2005; Ito *et al*., 2006). There is also a reduction in total HDAC activity in peripheral lung, bronchial biopsy specimens, and alveolar macrophages from COPD patients, and this is correlated with disease severity and with increased gene expression of IL-8 (Ito *et al*., 2005). HDAC activity is reduced in alveolar macrophages of cigarette smokers compared to nonsmokers, and this is correlated with increased expression of inflammatory genes in these cells (Ito *et al*., 2001). Importantly, HDAC-2 has been found to mediate the deacetylation of the GR that enables NF-κB suppression (Ito *et al*., 2006). It was suggested that HDAC-2 is a key enzyme involved in the suppression of NF-κB-mediated inflammatory gene expression. Therefore, HDAC-2 reduction is involved both glucocorticoid-resistance and NF-κB-mediated inflammatory gene expression. The importance of this mechanism in glucocorticoid-insensitive COPD disease is emphasized by over-expression of HDAC-2, which restores glucocorticoid sensitivity in primary cells from these patients. The reasons for the reduction in HDAC, particularly HDAC-2, in COPD are not yet completely understood. However, there is increasing evidence that this may be due to inactivation of the enzyme of nitro-oxidative stress, in particular ONOO^–^ (Marwick *et al*., 2004; Moodie *et al*., 2004; Rahman *et al*., 2004).

Interestingly, it was reported that the bronchoalveolar lavage cellular constituents of patients with SM-induced asthma and chronic bronchitis (most frequent delayed lung toxicities) are similar to those that have been observed previously in patients with asthma and chronic bronchitis from other common causes (Emad and Rezaian, 1999). They also revealed a number of pathophysiological similarities between SM-induced lung toxicity and pulmonary fibrosis (Emad and Emad, 2007) as well as bronchiectasis. Therefore, it is speculated that SM-induced delayed toxicity may be mediated by epigenetic perturbations at least in lung tissue. Further experimental studies are needed to clarify the pathophysiological mechanism.

## Possible Beneficial Effects of Melatonin Against Delayed SM Toxicity

Melatonin shows beneficial effects against SM-induced acute toxicity as a multifunctional antioxidant and ONOO^–^ scavenging agent in both *in vivo* and *in vitro* (Sourdeval *et al*., 2006; Ucar *et al*., 2007). Also, several well-explained effects of melatonin seem to derive from epigenetic actions of the indolamine. For example, melatonin possesses genomic actions and regulates the expression of several genes. Melatonin influences cellular mRNA levels for antioxidant enzymes under both physiological conditions and during elevated oxidative stress (Rodriguez *et al*., 2004). Consistent evidence suggests that melatonin modulates antioxidant enzyme activities via interaction with calmodulin, which in turn modulates epigenetic activation leading to gene expression (Tomas-Zapico *et al*., 2005; Tomas-Zapico and Coto-Montes, 2005). A number of known anti-inflammatory effects of melatonin, such as selective inhibition of iNOS and/or cyclooxygenase-2 and MMPs clearly derive from melatonin and epigenetic cross-talk and modification through suppression of NF-κB binding (Esposito *et al*., 2008) and/or p300-HAT expression within the nucleus (Deng *et al*., 2006). The action of melatonin in advanced cancer patients (Lissoni *et al*., 2001) also seems to result from a combination of effects on histone modification and DNA methylation (Cui *et al*., 2006; Korkmaz and Reiter, 2008). Recently, direct evidence of epigenetic actions for melatonin including nuclear receptors, co-regulators and histone acetylating enzymes has been reported (Sharma *et al*., 2008). In this study, melatonin significantly increased mRNA expression for various HDAC isoforms and increased histone H3 acetylation in neural stem cell lines.

## Concluding Remarks

Despite 75 years of research, there is still no antidote for mustard. This fact is especially crucial when we consider that probably at least a dozen countries have mustard in their arsenals today. Melatonin has been administered in both physiological and pharmacological amounts to humans and animals, and there is widespread agreement that it is a non-toxic molecule. In pregnant rats, maternal lowest no observed effect level has been found to be 200mg/kg/day and developmental no observed adverse effect level is ≥ 200 mg/kg/day (Jahnke *et al*., 1999). Melatonin is easily synthesized in pharmacologically pure form, non-patentable, inexpensive and affordable; therefore, it has a great potential to improve the public health (Reiter, 2006) as a multi-tasking molecule. Melatonin has non-genomic, genomic and epigenetic actions; all these actions may be beneficial in both acute and delayed mustard toxicity.
